# Colony life history of the tropical arboreal ant, *Cephalotes goniodontus* De Andrade, 1999

**DOI:** 10.1007/s00040-024-00974-3

**Published:** 2024-06-27

**Authors:** I. A. E. Butler, T. Butterfield, M. Janda, D. M. Gordon

**Affiliations:** 1 Instituto de Biología, Universidad Nacional Autónoma de México, 04510 Mexico City, Mexico; 2 Estudiantes Conservando La Naturaleza AC, 85760 Alamos, Sonora Mexico; 3 Laboratorio Nacional de Análisis y Síntesis Ecológica, Escuela Nacional de Estudios Superiores Unidad Morelia, Universidad Nacional Autónoma de México, 58190 Morelia, Michoacán Mexico; 4 Department of Zoology, Faculty of Science, Palacky University Olomouc, Olomouc, Czech Republic; 5 Biology Centre of Czech Academy of Sciences, Institute of Entomology, Branisovska 31, 37005 Ceske Budejovice, Czech Republic; 6 Department of Biology, Stanford University, Stanford, CA 94305 USA; 7 Instituto de Investigaciones Biomédicas, Universidad Nacional Autónoma de México, 04510 Mexico City, Mexico

**Keywords:** Arboreal ants, *Cephalotes*, Microsatellites, Relatedness

## Abstract

Arboreal ants are ecologically important in tropical forests, but there are few studies using DNA markers to examine their population and colony structure. Colonies of the arboreal turtle ant *Cephalotes goniodontus* create trail networks through the canopy of the tropical forest, in dense vegetation where it is difficult to determine how long a nest is used and how neighboring colonies partition space. We monitored 53 nest sites for up to six years and, using seven microsatellite markers, genotyped samples of workers collected at or near 41 nests over 1–4 years. We calculated average relatedness within samples collected at a given location, and between samples collected at the same location in successive years, and performed pedigree analysis to predict the number of queens that produced each sample of workers. Fifteen samples were highly related (*r* ≥ 0.6) from single colonies, of which 11 were monogynous and the remaining four had two queens; 19 were of intermediate relatedness (0.1 ≤ *r* < 0.6) with 1–6 queens, and 7 were groups of unrelated workers (r < 0.1) from at least 4 queens. Colonies persisted at the same nest site for 2–6 years. The smallest distance we found separating nests of different colonies was 16.2 m. It appears that different colonies may share foraging trails. Our study demonstrates the feasibility of using a cost-efficient genotyping method to provide information on colony structure and life history of ant species.

**Supplementary Information:**

The online version contains supplementary material available at 10.1007/s00040-024-00974-3.

## Introduction

Ants are enormously important in the ecology of tropical forests. They perform many crucial ecological functions, as consumers of nectar, insects, and of leaves to cultivate fungus, and they act as mutualist partners that deter the herbivores of many plants. Although the number of studies on ant population biology is growing, there are still few studies on the population biology of arboreal ants, which are abundant in tropical forests (Davidson and Patrell-Kim 1996; Longino and Colwell 2020). Basic questions about colony life history and population dynamics remain unanswered because it is difficult to identify and track individual colonies, which can occupy many hidden nests (Debout et al. 2003; Miranda et al. 2021; Powell and Peretz 2021) that may change position frequently (Rastogi 2007).

Most studies of arboreal ants rely on indirect sampling methods, such as baits and fogging (Floren and Linsenmair 2000; Yanoviak and Kaspari 2000; Blüthgen and Feldhaar 2010; Philpott et al. 2014; Longino and Colwell 2020; Leponce et al. 2021a). Recent studies provide new insight into the structure of arboreal ant assemblages, using direct sampling of felled trees (Klimes et al. 2012; Klimes and McArthur 2014; Klimes et al. 2015; Mottl et al. 2019), tree-climbing techniques, and complementary methods (Powell et al. 2011; Camarota et al. 2016; Dejean et al. 2018; Leponce et al. 2019; Leponce et al. 2021a; Leponce et al. 2021b). However, these methods provide only limited data on the life history of particular colonies and on population dynamics.

Previous work on the population and social structure of arboreal ants, and interactions among neighboring colonies, used aggression at baits, among workers from different trees, to estimate the boundaries of the foraging areas of particular colonies (Maeyama and Matsumoto 2000; Debout et al. 2003; Rastogi 2007; Mathis et al. 2016; Dejean et al. 2019). Although there is evidence that colonies of some arboreal species maintain exclusive foraging areas, it is not known how dominance at baits reflects all of the processes that determine community structure.

Valuable insight into the spatial distribution and population structure of arboreal ants has been provided by examining mutualistic species associated with myrmecophytic plants and epiphytes (Debout et al. 2003; Frederickson and Gordon 2009). The spatial distribution of colonies in a population depends on the location of available nest sites (Carroll 1979; Philpott and Foster 2005; Powell et al. 2011). For example, the distribution of epiphytes can facilitate ant diversity (Orivel and Leroy 2011) or facilitate a discrete, non-overlapping distribution of particular species (Volp and Lach 2019).

While many studies of ground-nesting ants have used DNA markers to examine population structure, only a few studies have done this to investigate the colony life history of arboreal ant species. Boyle et al. (2018) showed that the competition among colonies of three *Crematogaster* species and *Tetraponera penzigi* nesting in *Acacia* was not associated with variation in polygynous colony structure and that the mating system varies considerably between these arboreal species with similar ecology. Using microsatellites, Eyer et al. (2021) inferred the social structure of the arboreal ant *Mellisotarsus,* showing that colonies have one actively reproducing polyandric queen and that genetically diverse colonies can inhabit a single tree. By contrast, *Myrmelachista schumanni* forms huge colonies, occupying hundreds of host trees. Colonies can be polygynous or monogynous with facultative polyandry, and surprisingly, there was no genetic structure detected among trees or patches of vegetation (Malé et al. 2020). Schlüns et al. (2009) used microsatellites to show that *Oecophylla smaragdina* colonies are primarily monogynous, but that queen mating frequency varies by locality. Apart from these few pioneering studies, little is known about the population genetic structure and life history of colonies of tropical arboreal ants.

*Cephalotes* is a large genus of arboreal ants, widespread in the neotropics (Powell 2008). The role of *Cephalotes* species in competition for nest sites has important effects on tropical ant community structure (Powell et al. 2011). Its phylogeny is well understood, reflecting its coevolution over 50 million years with nitrogen-recycling bacteria, originating in the South American Cerrado region (Russell et al. 2009; Sanders et al. 2014; Graber et al. 2023). Its microbiome allows it to consume nitrogen sources and carbohydrates, such as pollen, lichens, nectar, homopteran secretions, and bird and lizard droppings (Baroni Urbani and De Andrade 1997; Davidson et al. 2004; Byk and Del-Claro 2010; Ramalho and Moreau 2023) without the need to consume protein directly. Colonies of many *Cephalotes* species tend to be founded by a single queen (De Andrade and Urbani 1999), though *C. atratus* is facultatively polygynous (Price 2011), as may be other species in the genus.

*Cephalotes goniodontus* De Andrade, 1999, distributed along the Pacific coast of central Mexico (Janicki et al. 2016), nests in tunnels created in decayed wood by larval beetles (Novais et al. 2017). The tunnels are often located in clusters within about 5 cm^2^, apparently reflecting the spatial distribution of eggs laid by the beetle. A colony uses all of the tunnels in a cluster as its nest. Colonies frequently add nests, on a timescale of weeks, by locating new larval tunnels at a different place in the vegetation, often many meters away, and lose nests when a decayed branch that a nest is in breaks and falls to the ground (Gordon 2017).

A colony of *Cephalotes goniodontus* creates a trail network that can extend at least 100 m, including many nest sites. The trail forms a circuit connecting all the nests with temporary trails to food sources (Gordon 2012; Gordon 2017), as in other *Cephalotes* species (Chang et al. 2021). The network is maintained by the flow of ants on the trail, as ants put down a volatile trail pheromone as they walk, and at each junction, where there is a branch in the vegetation or a choice of paths, ants are likely to take the path most reinforced by the previous ants through that junction (Chandrasekhar et al. 2018; Chandrasekhar et al. 2021; Garg et al. 2023). Because the trail network of a single colony can spread over many tens of meters (Gordon 2012), it is difficult to determine what are the boundaries of a colony’s foraging area and how long a colony lives. Colonies of *C. goniodontus* appear to persist at the same site from year to year (DMG pers. obs.). It is not known for any *Cephalotes* species how long a colony lives and whether neighboring colonies are close enough to compete for resources.

We monitored nests of the arboreal turtle ant *C. goniodontus* for 6 years, 2016–2021, at the Estación de Biología Chamela in Jalisco, Mexico. Here we used population genetic methods to address several questions:

1. Does the same colony occupy the same site from year to year?
2. How long does a colony survive?
3. How close are neighboring colonies to each other?
4. Is there any evidence that ants of neighboring colonies may forage along the same trails?

## Methods

### Study site

The study was conducted at the Chamela Biological Station (Chamela) of the National Autonomous University of Mexico, near Chamela, Jalisco, Mexico (19°30′ N, 105°03′ W). The tropical dry forest at Chamela has a prolonged dry season, with a total annual precipitation of 757 mm concentrated between June and October but extended into November when the hurricane season ends (rainy season mean = 668 mm; dry season mean = 105 mm; [Bullock 1986; García-Oliva et al. 2002; Lott and Atkinson 2002]). The mean annual temperature is 25 °C, with small variation throughout the year due to the moderating effect of the ocean (Hayden et al. 2010). The vegetation is a tropical deciduous forest with a dominant woody layer and mainly deciduous tree species that are on average 15 m high. Most trees are leafless between December and May (Cortés‐Flores et al. 2023).

### Monitoring nests

We located nests of *C. goniodontus* from 2016 to 2021 by walking along the paths in the forest and searching for ants in the vegetation. When an active trail was found, we followed the trail until we saw the ants entering a nest hole in a branch. We followed trails as far as possible to search for other nests of the same colony as in previous work (Gordon 2017). We recorded nest locations using a Garmin handheld GPS receiver. We checked each nest and the area around it at least once per year. We considered a nest to be active on a particular day if ants were seen entering or exiting near the nest entrance, and inactive if no ants were observed at the nest or in the surrounding vegetation in at least 2 visits in a given year.

### DNA genotyping

**Fig. 1:**
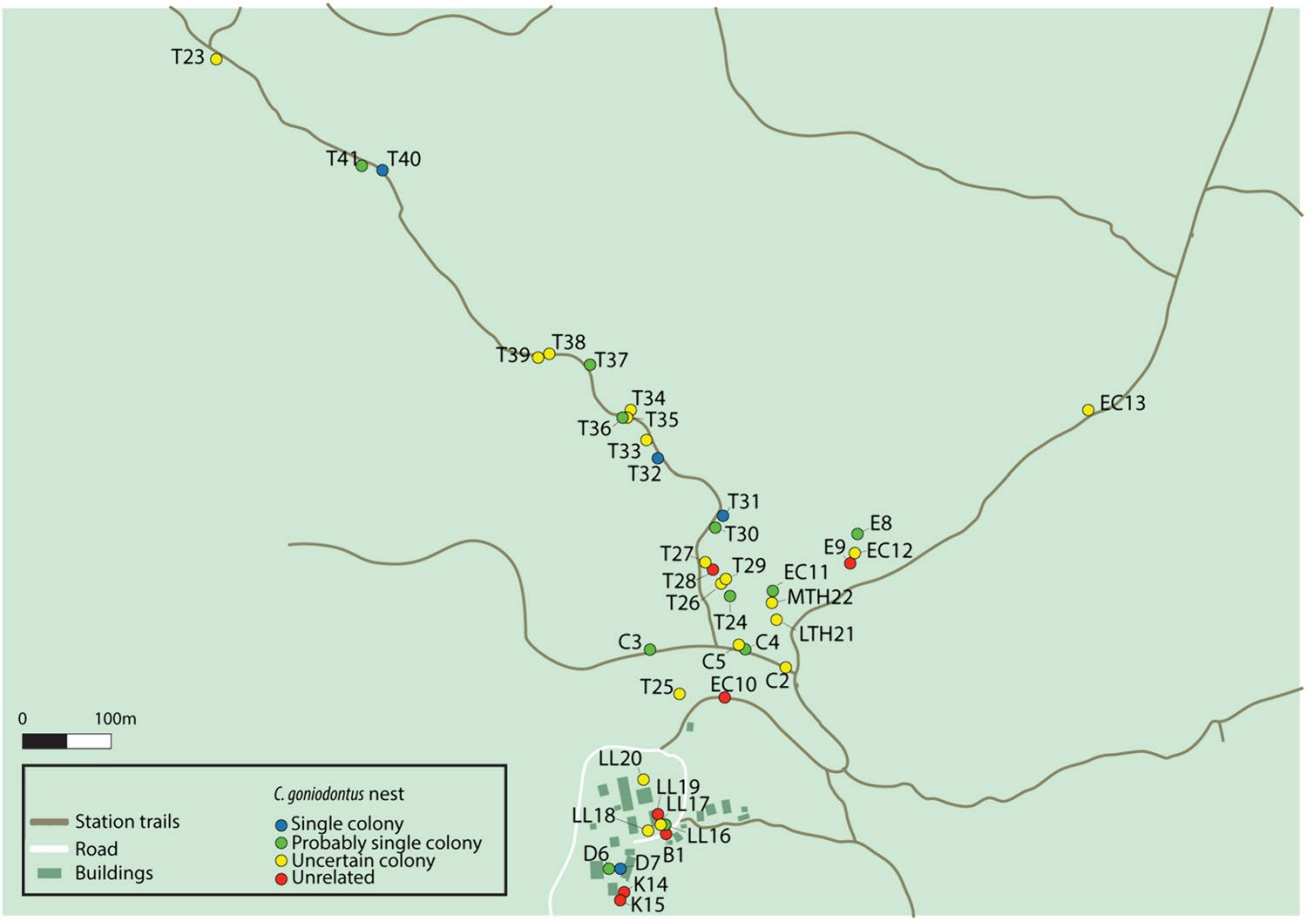
Map of sampling sites at the Chamela Biological Station. Each circle corresponds to a sampling site, and the color of the circle represents the relatedness category assigned to samples from that site. The four categories correspond to different levels of mean pairwise relatedness within the nest (Category 1 (blue): single colonies, mean relatedness within the colony ≥ 0.6 and all pairwise relatedness values ≥ 0.5; Category 2 (green): probably single colonies, mean relatedness ≥ 0.6; Category 3 (yellow): uncertain colonies, mean relatedness < 0.6 and ≥ 0.1; Category 4 (red): unrelated, mean relatedness < 0.1) (color figure online)

From 2017 to 2020 we collected samples of 10–30 ants as they entered an active nest or from a foraging trail apparently near a nest entrance (Fig. 1). All sampling was done at the nest if it was accessible, or as near to a nest as it was possible to reach (as some nests were high in the trees); sampling was not done along the foraging trails which can extend for tens and even hundreds of meters (Gordon 2012, 2017). Here we refer to the place where a sample was collected, either at or near a nest, as a ‘site’. Specimens were preserved in 95–99.8% ethanol and DNA was obtained from 1 to 20 individuals from each sample. Total genomic DNA was extracted from up to three legs or a whole individual using the Genomic DNA Kit (Geneaid, South Korea). A set of seven microsatellite loci were selected from a set of universal ant microsatellite primers previously developed by Butler et al. (2014). Microsatellites were chosen from 30 potential primer pairs based on high variability in a priori tests in *C. goniodontus* workers from different sites in the forest at Chamela. The list of loci can be found in Table S1.

The PCR cocktail (10 µl total volume) contained 4 µl of Master Mix Qiagen Multiplex PCR, 1 µl of Q solution, and 2 µl of DNA template. The PCRs were multiplexed in two sets. One set had three primer pairs (Ant859, Ant11315, and Ant11893) with 0.33 µl of each forward and reverse primer. The other set had four primer pairs (Ant4155, Ant1368, Ant8498, and Ant8424) with 0.25 µl of each forward and reverse primer. Water (1 µl) was added to bring the total volume of each reaction to 10 µl. The PCR reaction was performed on a BIORAD thermal cycler under the following conditions: 15 min at 95 °C, followed by 35 cycles of 94 °C for 30 s, 50 °C for 90 s, and 72 °C for 60 s and a final extension of 30 min at 72 °C (Butler et al. 2014). PCR products were visualised on agarose 1.5% to verify fragments. Samples were sequenced by Macrogen Inc. using the LIZ500 matrix, and the size of the loci was estimated in the software Geneious Prime 2019–2021. All alleles were scored by a single person and checked twice. Before scoring, all microsatellites were checked for consistency between individuals. Inconsistent loci were not scored and excluded from analyses.

### Summary statistics

Social insect colonies are composed of related individuals, and this may influence estimates of allele frequencies in a population. To account for this, we randomly selected one worker from each nest to calculate population allele frequencies. This subsampling method was repeated three times (hereafter subsampling 1, subsampling 2, and subsampling 3) to allow for sampling error. All subsequent analyses are repeated three times, once for each of the subsampling schemes. Observed and expected heterozygosities were calculated using GenAlEx v6.5 (Peakall and Smouse 2006; Peakall and Smouse 2012). Tests for deviations from Hardy–Weinberg equilibrium (HWE) and linkage disequilibrium (LD) were performed using exact tests implemented in Genepop on the Web v4.7.5 (Raymond and Rousset 1995; Rousset 2008).

### Genetic relatedness

We estimated pairwise relatedness (*r*, Queller & Goodnight, 1989) values for 912 individuals at 41 sites collected between 2017 and 2020 using the software package SPAGeDi v1.5d (Hardy and Vekemans 2002). We used the results for pairwise relatedness to: (i) evaluate whether ants collected at a particular nest site represented a single colony, (ii) estimate whether samples from different nearby sites were from the same colony in a given year, and (iii) determine whether samples from the same site in different years were from the same colony.

To determine whether ants collected from a single site were from a single colony, we divided the samples into four categories based on mean pairwise relatedness within the nest. We did this for each year’s sample separately, and then, to ask whether the same colony persisted at a site over the years, we pooled relatedness values from all years together. The categories were:

1. Single colonies. Mean *r* ≥ 0.6 and pairwise *r* ≥ 0.5. Samples with mean relatedness greater than or equal to 0.6 and with all pairwise relatedness values within the sample greater than or equal to 0.5. Expected relatedness for workers that are full siblings is 0.75, so these samples are likely from monogynous colonies.
2. Probably single colonies. Mean *r* ≥ 0.6. Samples with mean relatedness higher than 0.6, but with some individual pairwise relatedness values lower than 0.5. These samples could be from a monogynous colony with some low relatedness values due to chance, could include unrelated workers from another colony, or could be from a polygynous colony.
3. Uncertain colonies. Mean *r* < 0.6 and ≥ 0.1. These samples may include members of the same colony and some unrelated individuals, or could be from a monogynous colony whose queen had a large number of mates.
4. Unrelated workers. Mean *r* < 0.1. Samples with mean relatedness less than 0.1. These samples are likely to include workers from different colonies.

The cutoff value of 0.6 to distinguish categories 1 and 2 from 3 was chosen by inspecting a histogram of all intranest pairwise relatedness values across all samples (Figure S1). This histogram showed two peaks, one centered around 0.75, probably representing full siblings, and a second centered around 0.125, which probably includes half-sisters (expected relatedness 0.25), but also includes lower relatedness values indicating individuals from other colonies that may or may not be related. The 0.6 cutoff for categories 1 and 2 was chosen as low enough to include most full sibling colonies with minimal overlap with the lower peak. We consider the cutoff values to be conservative. Samples in category 3 probably contain both samples of ants from the same colony with a multiply-mated queen, as well as groups of unrelated workers.

In some cases, samples with relatedness values near one of the cutoff values were in different categories in analyses from different subsampling schemes. In these cases, to obtain a conservative estimate of the number of samples in each category, we assigned the sample to the category with lower relatedness (e.g. a sample in category 1 in subsampling 1 and in category 2 in subsampling 2 was considered to be in category 2).

We genotyped ants from 13 sites in 2017, 26 sites in 2018, 24 sites in 2019 and 18 sites in 2020. In total, we analysed samples from 43 sites, collected over four years, with a total of 912 workers. There were 24 sites for which samples were collected in more than one year. Of these, 13 were collected over 2 years, 7 over 3 years, and 4 over 4 years. For two samples (the 2018 sample from CN3 and the 2019 sample from E9), data were excluded from analyses where years were considered independently because only two workers were genotyped for that year. To evaluate whether a colony persisted at the same site year after year, we used a Wilcoxon rank sum test to determine if the average relatedness between a pair of years was significantly lower than the average relatedness within years. A Bonferroni-Holm correction was used to account for multiple comparisons.

### Pedigree analysis

To confirm our assignment of each sample to each relatedness category, we used the pedigree analysis software COLONY v2.0.7.0 (Jones and Wang 2010) to predict the number of parent queens and males represented in each sample, based on the worker genotypes. Each sample was run separately, with options for male monogamy, female polygamy, haplodiploidy, and dioecy turned on. We applied sibship scaling, and the sibship size prior was set to ‘very strong’ with the predicted sibship size for both maternal and paternal sibships set to the number of workers in the sample. The length of each run was ‘very long’ and each run was replicated 3 times. Allele frequencies were set to ‘known’ and were not updated based on inferred relationships, with allele frequencies calculated from subsampling 1 (see Results) used as input.

## Results

### Sampling statistics

We used 3 subsampling schemes to evaluate allele frequencies in the 912 workers collected over 4 years from 43 sites. For all three subsampling schemes, allele frequencies at all loci were consistent with Hardy–Weinberg expectations (Table S1), and no loci were linked, with the exception of Ant11893 and Ant4155, which were linked only in subsampling 3 (Table S2). The characteristics of the loci are shown in Table S3.

The results from all three subsampling schemes were identical in their outcomes, with two exceptions: in one case, the outcome was significant in subsampling 1 and 2 but not in subsampling 3, and in the second, the outcome was significant only in subsampling 2 (see next subsection). All results reported here from further analyses are from subsampling 1. Results from subsampling 2 and 3 are shown in Table S4.

### Genetic relatedness

We calculated the average pairwise relatedness for all samples. When the average relatedness from a sample was considered separately for each year, there were 10 samples in category 1, 20 in category 2, 38 in category 3 and 10 in category 4. When the samples from a single site for all years were pooled, there were 4 single colonies in category 1, 11 probable single colonies in category 2, 19 uncertain colonies in category 3, and 7 unrelated samples in category 4 (Table 1 and Table S5).

**Table 1:** Summary of number of samples in each category

	Mean *r* > 0.6, all individuals *r* > 0.5	Mean *r* > 0.6	Mean 0.6 > *r* > 0.1	Mean *r* < 0.1
All years pooled	4	11	19	7
Years evaluated separately	10	20	38	10

**Fig. 2:**
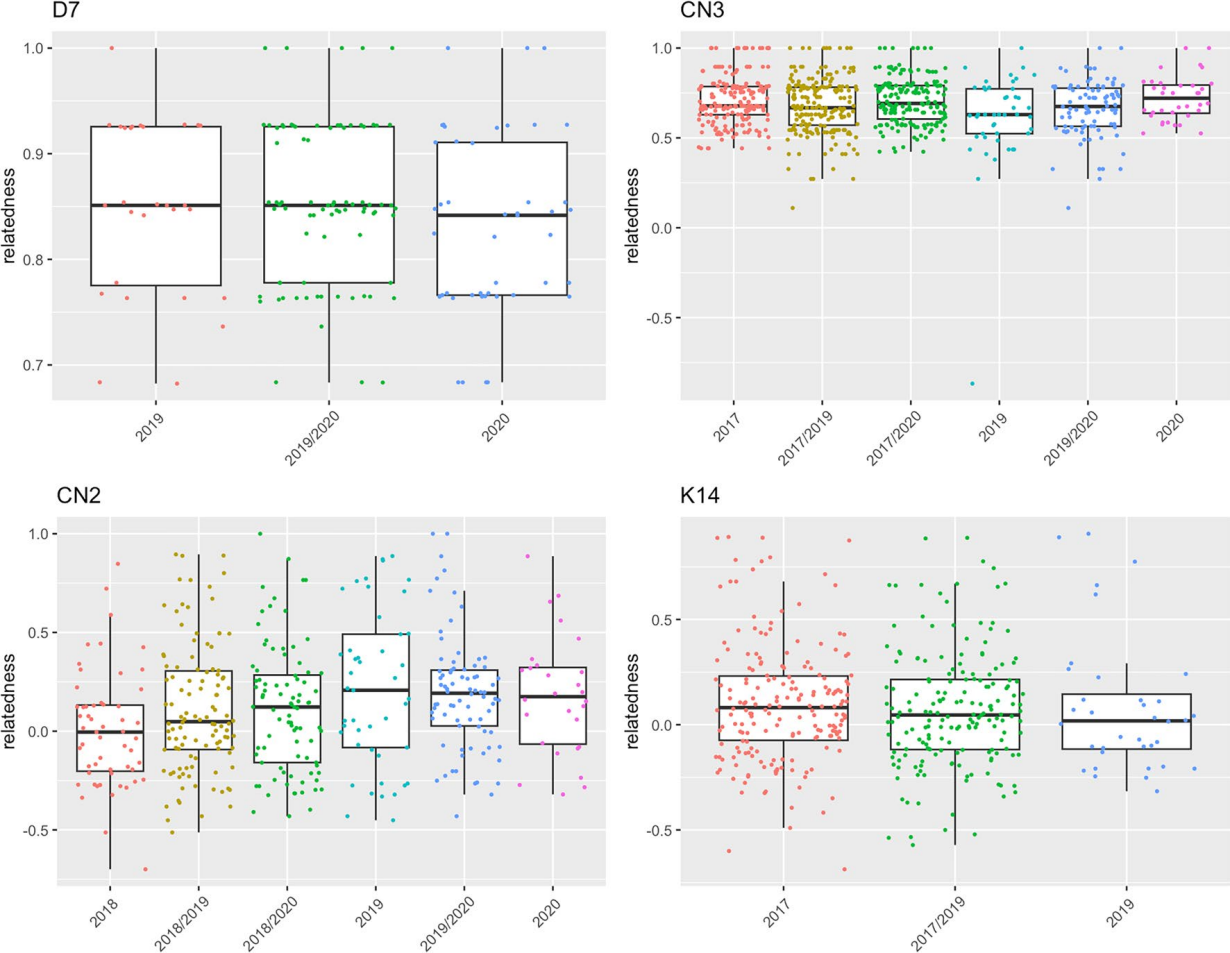
Boxplots showing the comparison of mean relatedness within years to relatedness across years, with one example from each relatedness category. Sample D7 is category 1 single colony; CN3 is category 2 probably single colony; CN2 is category 3 uncertain colony; K14 is category 4, unrelated. Relatedness values for all other samples are shown in Figures S2–S5

**Fig. 3:**
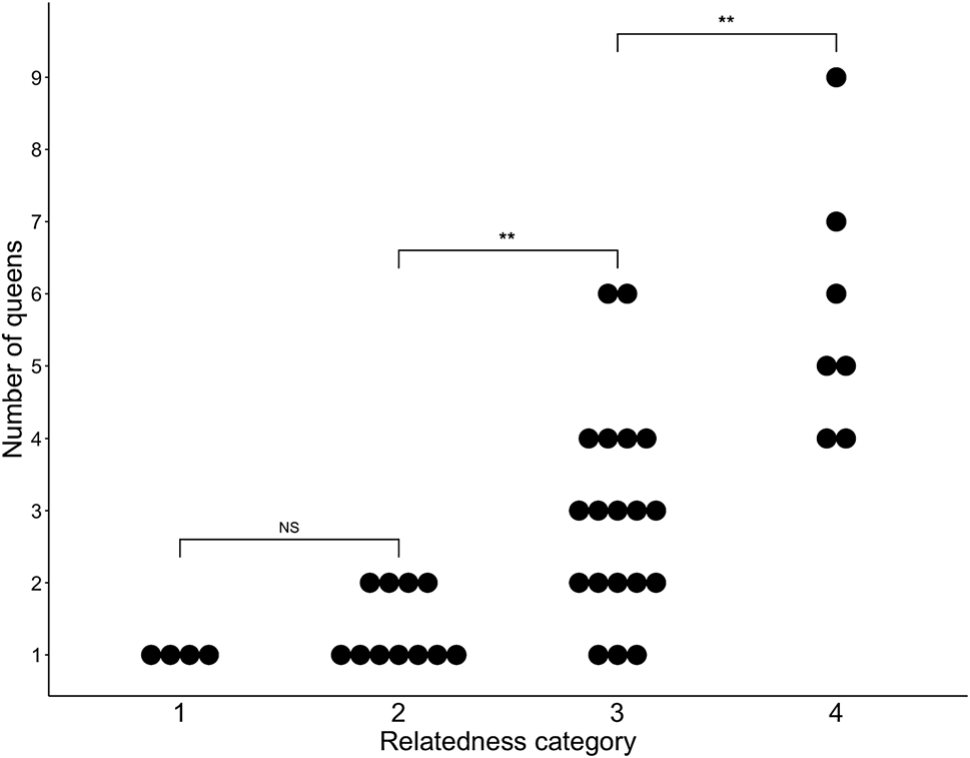
Number of queens assigned by COLONY by relatedness category (categories 1 and 2 probably a single colony; 3 maybe a single colony; 4 unrelated workers from different colonies). Asterisks indicate significant differences at *p* < 0.01 (**); NS = not significant (Wilcoxon rank sum tests, Bonferroni-Holm correction for multiple comparisons)

We compared samples collected at the same site in different years. For most samples there was no significant difference between intra-year and inter-year relatedness for any year. This was the case for both apparent colonies with multiple years of data in category 1, 7 of 8 probable colonies in category 2, 11 of 12 uncertain colonies in category 3, and 1 of 1 unrelated sample in category 4 (Fig. 3, Figures S2 -S5). Figure 2 shows data for one representative set of samples in each relatedness category.

There were 3 samples from the same site in more than one year that showed significantly lower inter-year than intra-year relatedness for some years. In T26 (category 3) inter-year relatedness between 2018 and 2019 was significantly lower than intra-year relatedness from 2018 (Figure S4) (Wilcoxon rank sum test, *p* = 0.01225 after Bonferroni-Holm correction). For T24 (category 2), inter-year relatedness between 2017 and 2018, 2017 and 2019, and 2017 and 2020 were all significantly lower than intra-year relatedness from 2017 (Figure S3) (Wilcoxon rank sum test, *p* = 0.0000003973, *p* = 0.001792 and *p* = 0.01365 after Bonferroni-Holm correction, respectively). The differences in T24 and T26 were significant for all subsampling schemes, with the exception of inter-year relatedness between 2017 and 2020 compared to intra-year relatedness from 2017, which was not significant in subsampling 3 (Table S4). Inter-year relatedness between 2018 and 2020 in sample EC11 was significantly lower than intra-year relatedness from 2018 for subsampling 2 only (Wilcoxon rank sum test, *p* = 0.04514 after Bonferroni-Holm correction) (Table S4).

### Pedigree analysis

Most of the samples with high enough relatedness to be from the same colony were from monogynous colonies (Fig. 3). The pedigree analysis by COLONY predicted a single queen in 4 of 4 samples in category 1 and 7 of 11 in category 2. As expected, the number of predicted queens increased significantly as relatedness decreased (Kruskall-Wallis rank sum test, *χ*^2^ = 25.254, df = 3, *p* = 0.00001366). In category 3, samples that may have come from the same colony, predicted queen number ranged from 1 to 6, while in category 4, samples with workers from different colonies, predicted queen numbers ranged from 4 to 9. Pairwise comparisons of results from each category showed no significant difference between categories 1 and 2 (*p* = 0.2021) and significant differences between 2 and 3 (*p* = 0.0038) and 3 and 4 (*p* = 0.0038) (Wilcoxon rank sum tests, Bonferroni-Holm correction for multiple comparisons).

Most queens were predicted to have more than one mate. This was found in all samples for which a single queen was predicted except for one (Table S6). In addition, in the category 2 samples for which 2 queens were predicted, all were multiply mated, except for three queens that were each assigned a very small number of workers (less than 4). By contrast, most samples from categories 3 and 4 with more than one queen predicted indicated only one mate was assigned to each queen, even queens with more than 10 assigned workers. The observed mating frequencies with standard error were: Category 1, 3.25 ± 0.479; Category 2, 3.00 ± 0.507; Category 3, 1.48 ± 0.119; Category 4, 1.68 ± 0.158. Because the number of mates is difficult to predict when the number of workers assigned to a particular queen is low, we also found the observed mating frequencies by category excluding queens with fewer than ten assigned workers: Category 1, 3.33 ± 0.667; Category 2, 3.73 ± 0.541; Category 3, 1.71 ± 0.339. No predicted queens in category 4 were assigned 10 or more workers.

### Colony longevity and spatial distribution

A total of 53 nest sites were monitored over 6 years between 2016 and 2021. (There were an additional 11 sites that were sampled in only one year for genotyping and were not monitored for more than one year). The number of years that activity lasted at the same nest site ranged from 1 to 6, with a mean of 2.4 (± 1.4 S.D.). Of the 53 nests, 23 were not sampled for genotyping. Most of these were seen in only one year and inactive the next. There were 20 nests seen in 1 year only, 14 for 2 years, 6 for 3 years, 7 for 4 years, 5 for 5 years, and 1 for 6 years. Of the 19 nests seen active for 3 or more years, only 1, seen for 4 years, was not sampled for genotyping.

Trails have been observed from one nest to another nest at least 20 m away (Gordon 2012, 2017, Chandrasekhar et al. 2021), but here we did not identify neighboring nests of the same monogynous colony occupied in the same year. There were two pairs of nearby nests occupied in different years, with low relatedness (category 3), so it is not certain if they were from the same colony. One set of samples, T23, had a mean relatedness of 0.545 overall, occupying one nest in 2017, 2018 and 2019, and a different nest 10.23 m away in 2020. Another set of samples, T27, had mean relatedness of 0.423 and occupied one nest in 2017 and a different nest 14.15 m away in 2018, 2019 and 2020. The closest nests of distinct colonies (categories 1 and 2) occupied in the same year were 16.2 m apart, between samples T30 and T31, both occupied in 2019 and 2020. Nests of samples D6 and D7 were 14.03 m apart, and nests of T40 and T41 were 23.54 m apart, but these neighboring nests were not simultaneously occupied. Nests of other distinct colonies that were simultaneously occupied ranged from 48.39 to 835.61 m apart, but there were probably other nests in between (Table S6).

## Discussion

Our results elucidate the population biology, spatial distribution, life history and foraging behavior of colonies of *C. goniodontus*. A colony can persist in the same area, and even in the same nest, year after year, up to at least six years. Our results comparing the relatedness of samples collected at the same site in more than one year support this conclusion. If a different colony tended to occupy a site each year, then we would expect to see higher relatedness within years than between years. The high relatedness within and between years strongly supports the conclusion that colonies persist in the same location for many years. Six samples with mean relatedness above 0.6 were clearly at the same site for two years (D6, D7, E8, LL16, T31, and T36), and two samples used the same site for three years (CN3, EC11, and T30), although EC11 had lower inter-year than intra-year relatedness for one comparison in subsampling 2. All but one of these samples (LL16) were predicted to be monogynous by the pedigree analysis, further supporting the conclusion that these were single colonies occupying the same site for 2 years or more.

There was further evidence, even in samples with lower relatedness, that colonies persisted at the same site for more than one year. For 12 of the other 14 sites sampled in more than 1 year, inter-year relatedness was the same as intra-year relatedness. This includes some nests with relatively high average relatedness (LL18, LTH21, T23, and T33) (Fig. 2, Figure S4), and two that showed consistent relatedness over four years (T23 and T27) (Figure S4). In one sample with mean relatedness above 0.6 that had lower inter-year than intra-year relatedness for some comparisons (T24), relatedness remained high in all inter-year comparisons, consistent with the conclusion this was a single colony. This conclusion is further supported by the result that relatedness was higher within than between years in only 1 of 4 years; if for this colony a sample from a particular year were from a distinct colony, relatedness should have been low for all inter-year comparisons. Pedigree analysis predicted that this sample had two queens with 6 and 4 mates, respectively (Table S6). Workers assigned to both queens were distributed throughout the sampled years, so this is unlikely to result from differences in sampled workers attributed to different queens in different years. The variation in relatedness values could be due to patriline shifting when the cohorts of eggs in each year differ in the proportion of offspring fathered by a particular male (Wiernasz and Cole 2010).

Some samples that showed low relatedness may have been from a single colony. For example, T33 has been observed in the same place, with the same nest, using very similar trails, each year from 2018 to 2022 (DMG, pers obs). Although it is in category 3, probably again due to the high variance in relatedness values, our pedigree analysis predicted a single twice-mated queen, and the observed relatedness (0.505) is consistent with a monogynous colony whose queen mated two times (minimum expected *r* = 0.5) (Bourke & Franks, 1995 p. 120).

It appears that many colonies are monogynous, as in other species of *Cephalotes* (De Andrade and Urbani 1999; Price 2011) (Table S6); this was indicated by pedigree analysis for most of the samples identified as a single colony. In addition, it seems likely that most queens of *C. goniodontus* are multiply mated. The high relatedness for some colonies is consistent with a colony in which the queen mated only once (category 1 and some colonies in category 2). However, pedigree analysis predicted multiple mates in all four category 1 samples and five of seven monogynous colonies in category 2. This discrepancy between the relatedness values and the pedigree analysis is probably due to the high variance in relatedness values. The mating frequencies estimated from categories 1 and 2 (3.33 and 3.73, respectively) are probably more accurate than those from categories 3 and 4, which had low relatedness and probably include ants from different colonies. Our observed mating frequencies may be overestimates if the workers sampled are actually from two or more related queens (full sisters or mother/daughter) with fewer mates. Our data do not allow us to distinguish between multiple queens with fewer mates and single queens with more mates, but as monogygny is prevalent in *Cephalotes* (De Andrade and Urbani 1999; Price 2011), we think that single queens are most likely. To our knowledge, this is the first study to measure mating frequency in any *Cephalotes* species. Future studies in this and other *Cephalotes* species are needed.

Our results suggest that different colonies share foraging paths and that one colony’s path may pass near the nest of another colony. In addition, it seems that trails of different colonies may sometimes intersect at the same place in the vegetation year after year, because some samples with low relatedness (categories 3 and 4) showed similar relatedness year after year. At the sites where we consistently found unrelated ants (K14), or found that after a gap of a year (E9, K14, and T34) the ants were not related to those of 2 years before, it may be that different colonies were consistently using overlapping trails in their respective trail networks. If nests persist in the same location for many years, then it is likely that the unrelated samples contained ants from the same distinct nearby colonies. The closest distinct colonies we found were only 16.2 m apart, while trail networks can extend for 100 m or more (Gordon 2012), so there are many opportunities for the trails of neighboring colonies to overlap. Some samples taken at the same site in more than one year included ants that probably came from different colonies, with mean relatedness values < 0.1 and many predicted queens. The possibility that neighboring colonies may be repeatedly using the same trails is especially intriguing because in 6 years of detailed mapping of foraging trail networks of the same colonies, day after day, no aggression between ants has ever been observed (Gordon 2017; Chandrasekhar et al. 2021). We once moved ants from the trail coming from one nest to a trail coming from another, in nests that we learned in this study were occupied by different colonies, but saw no aggression. It is possible that neighboring colonies with related queens show reduced aggression among non-nestmates that share foraging trails. It is not known how far queens disperse in this species.

The use of a volatile trail pheromone to determine the path of ants at each junction may encourage neighboring colonies to use overlapping trails in two ways. First, the ants of one colony may respond to the trail pheromone of another, as ants from one colony arrive at a junction recently used by ants of the other. At least some *Cephalotes* species also utilize foraging pathways of other ant species, such as *Azteca* or *Crematogaster*, and can engage in parabiotic associations with them (Adams 1990; Longino 2000; Powell et al. 2014). Second, the vegetation itself may lead ants of neighboring colonies to converge on the same path. The vegetation is tangled, creating high connectivity among paths; we estimated that from any branch off the trail, a path of 2–4 junctions leads back onto the trail (Table 1 in Chandrasekhar et al. 2021), so two colonies with nearby paths are likely to find a path that intersects both. In addition, the physical configuration of a junction in the vegetation makes some paths more likely to be used (Chandrasekhar et al. 2021, Garg et al. 2023). Junctions on large branches are more easily reinforced because successive ants are likely to take the same path, while junctions involving a tangle of possible paths, or shifting vegetation, are less likely to be reinforced because successive ants are unlikely to take the same path. Because junctions on large branches are more likely to be reinforced by ants of any colony, and large branches are more likely to persist from year to year than smaller vines, it may be that some larger branches create sites that channel ants of neighboring colonies onto the same path year after year.

Our results here open the way to examine variation among colonies of *C. goniodontus* in behavior and to learn how a colony's behavior changes over its ontogeny. This will make it possible to examine the population dynamics of this species of arboreal ant and to learn how natural selection is shaping variation among colonies in behavior.

## Supplementary Information

Below is the link to the electronic supplementary material.

Supplementary file1 (DOCX 3996 KB)

## Data Availability

The relatedness dataset is available in the Stanford Digital Repository. 10.25740/cc037jw0373
