# Supplementary material for: Colony life history of the tropical arboreal ant, Cephalotes goniodontus De Andrade, 1999
Source: Insectes Soc. 2024 Jun 27;71(3):271–81. doi: 10.1007/s00040-024-00974-3 (PMC11401787; doi:10.1007/s00040-024-00974-3)
Supplement: Supplementary file 1 — Supplementary file1 (DOCX 3996 KB) [file 40_2024_974_MOESM1_ESM.docx]

**Supplementary Information for Butler et al. 2024**

Figure S1. Histogram of all pairwise relatedness values.


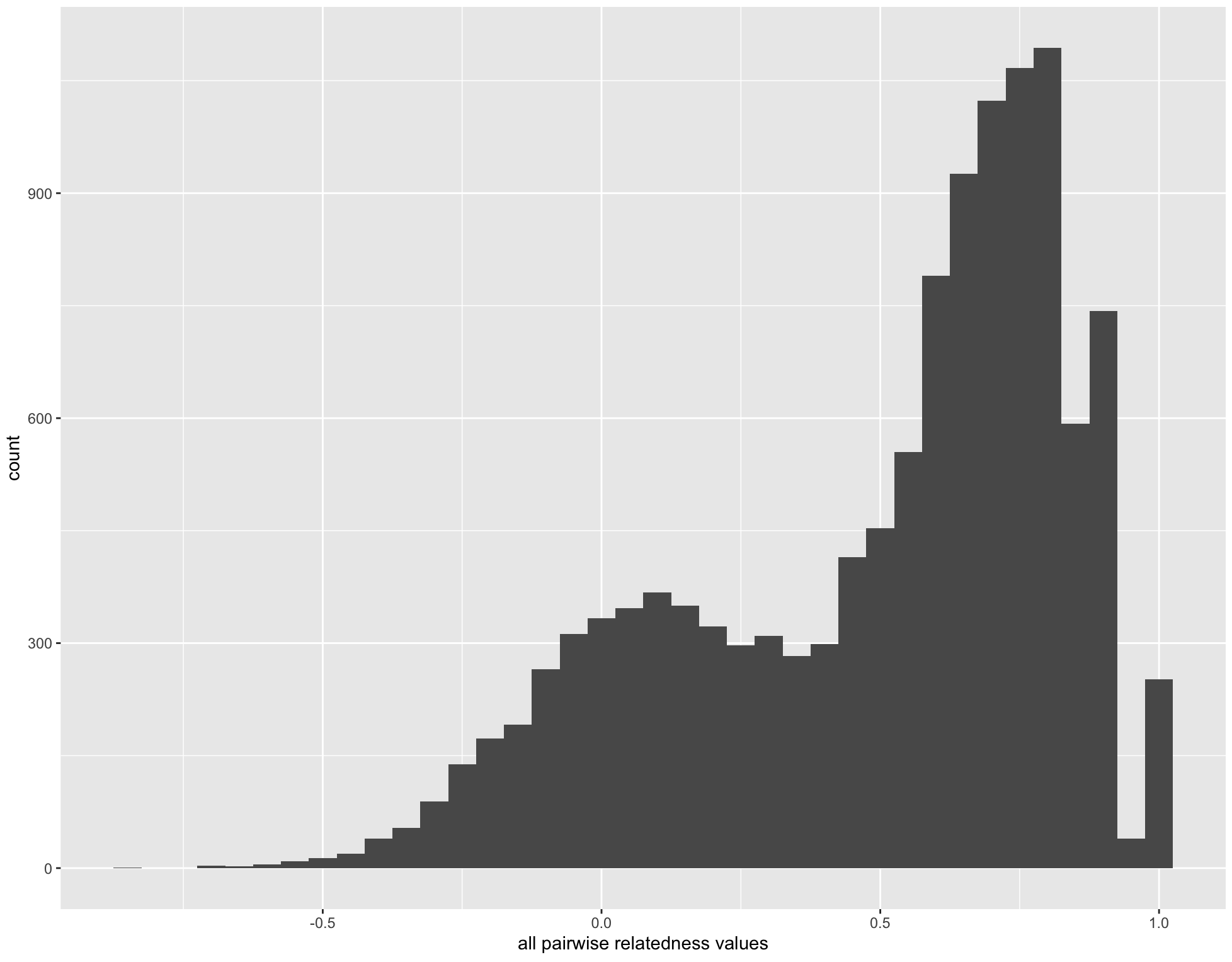


Figure S2. Relatedness boxplot of nest in category 1 not displayed in Figure 2.


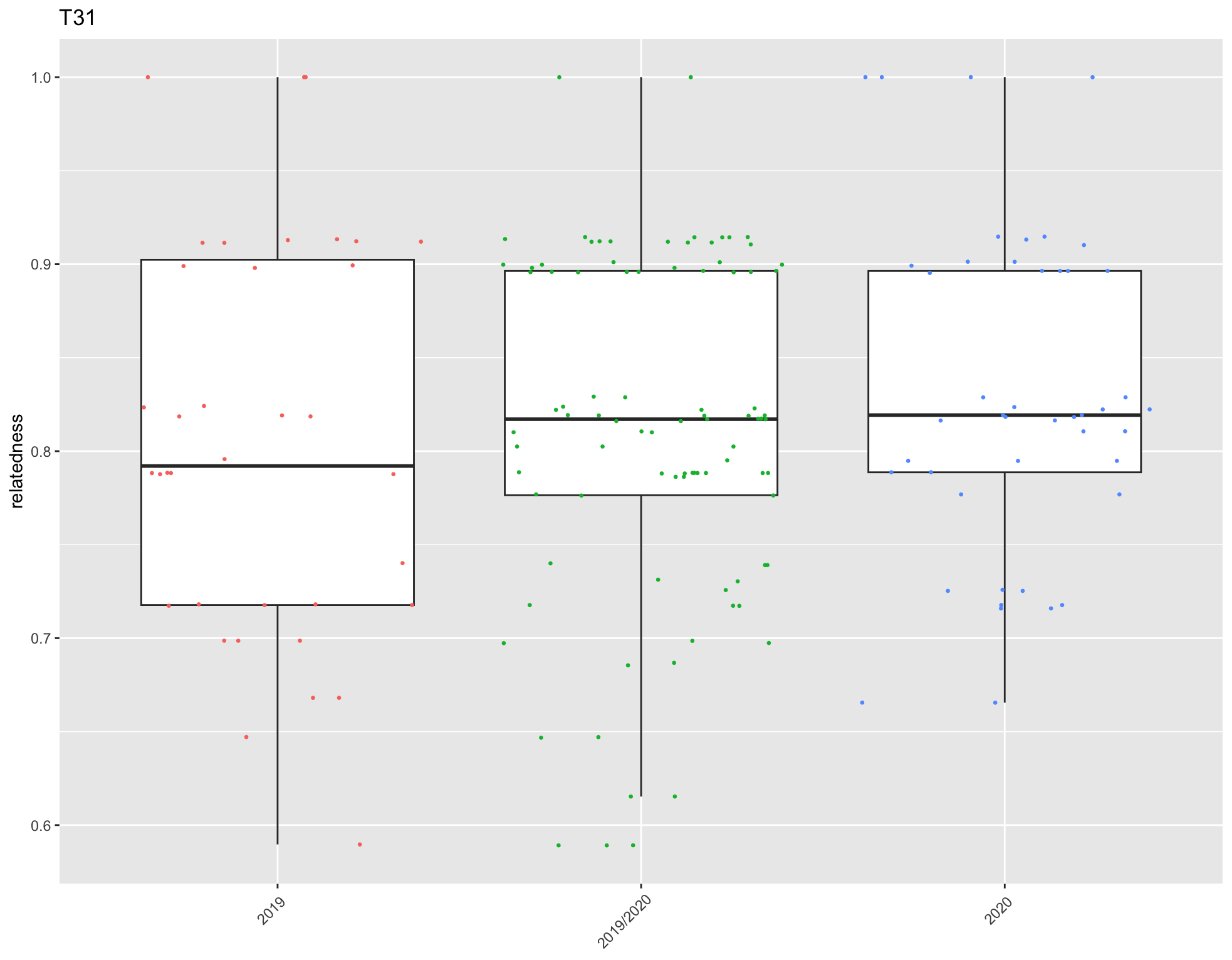


Figure S3. Relatedness boxplots of nests in category 2 not displayed in Figure 2.


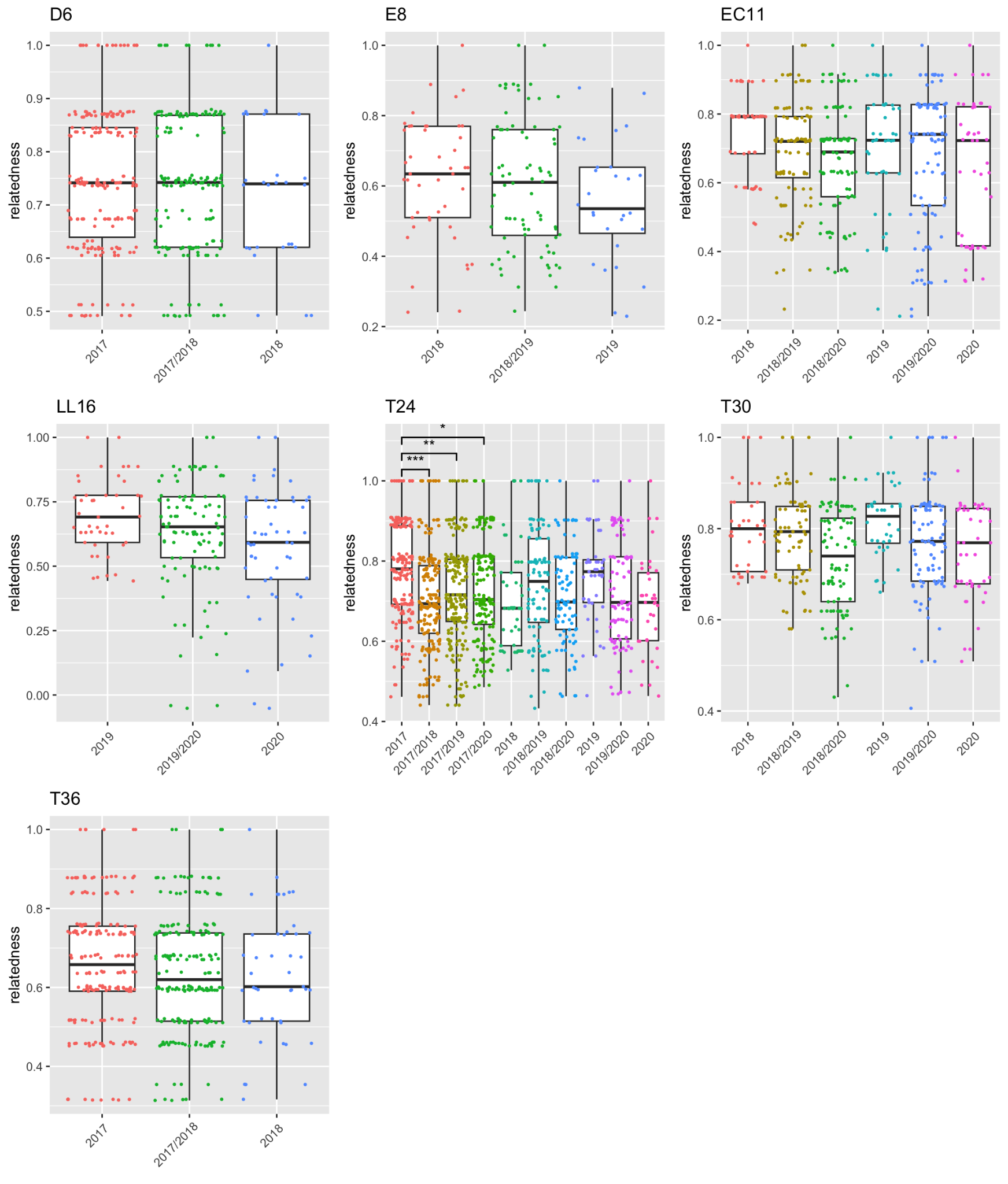


Figure S4. Relatedness boxplots of nests in category 3 not displayed in Figure 2.


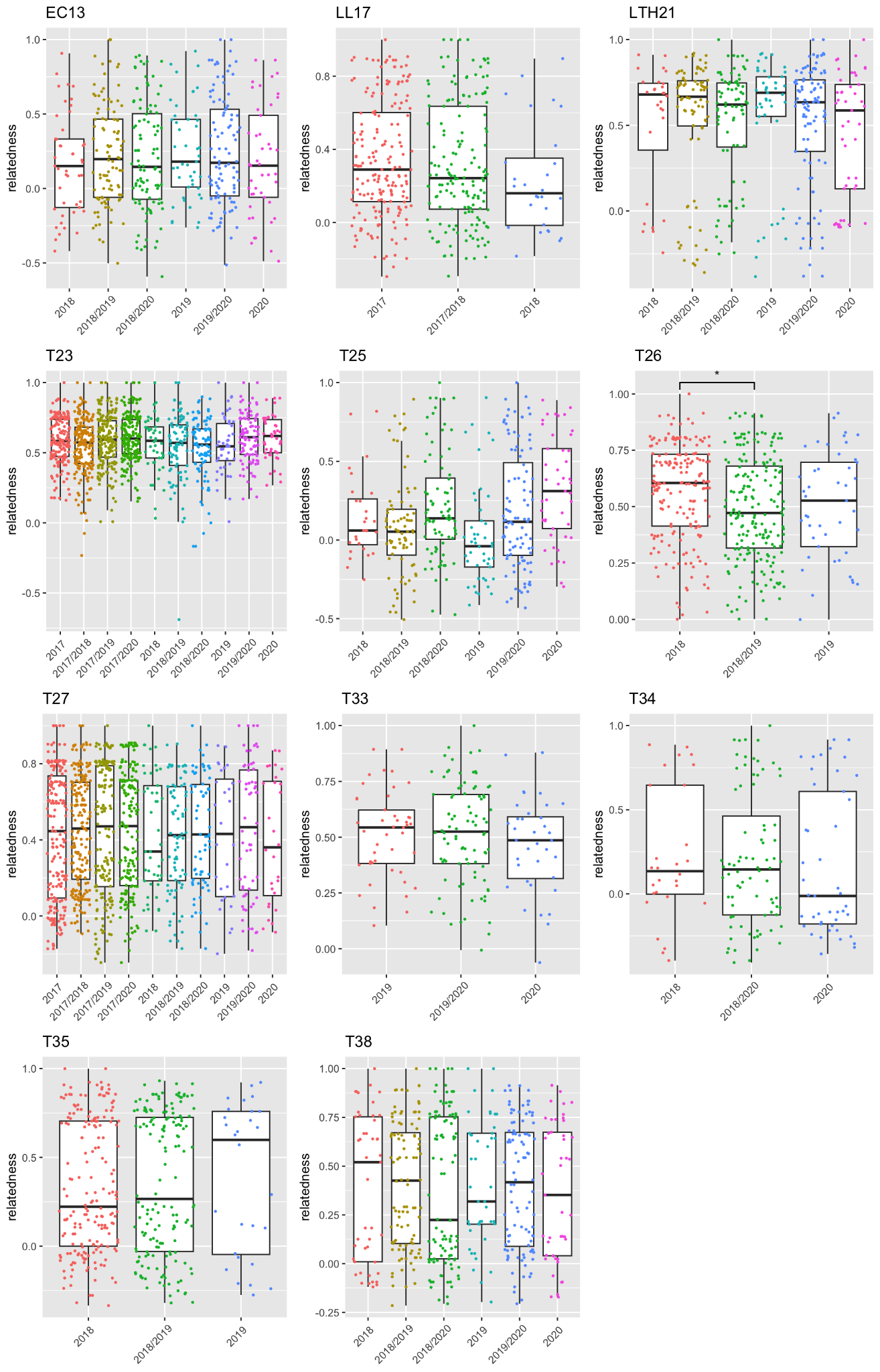


Figure S5. Relatedness boxplots for nests with only a single year of data. Nest E9 is included here because the 2019 data was dropped due to only two workers being genotyped for that year, leaving only one year of data.


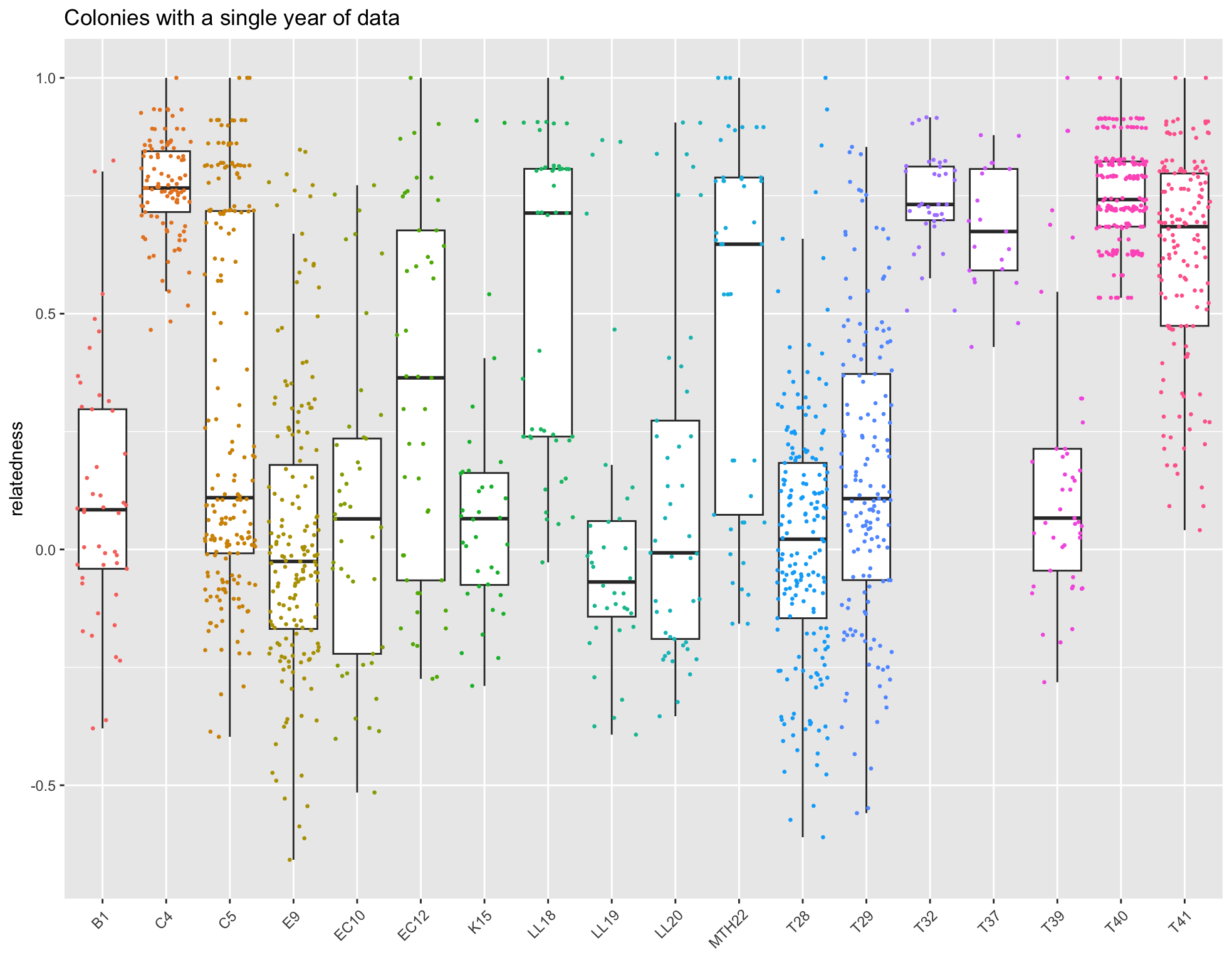


Table S1. Results of exact tests for Hardy Weinberg Equilibrium for each locus in each subsampling scheme calculated using Genepop on the Web v4.7.5 [(Raymond and Rousset 1995; Rousset 2008)](https://www.zotero.org/google-docs/?tmR1a2). Fis is estimated according to [Weir and Cockerham](https://www.zotero.org/google-docs/?P72NwA) (1984). Switches refers to the number of switches performed in the Markov Chain during the calculation of the P-value.


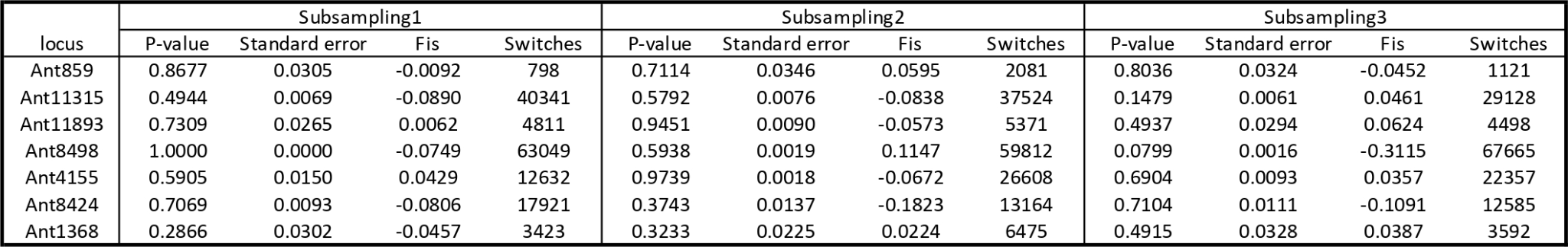


Table S2. Results for exact tests of linkage disequilibrium for all pairs of loci in each subsampling scheme calculated using Genepop on the Web v4.7.5 [(Raymond and Rousset 1995; Rousset 2008)](https://www.zotero.org/google-docs/?nyADvB). Switches refers to the number of switches performed in the Markov Chain during the calculation of the P-value.


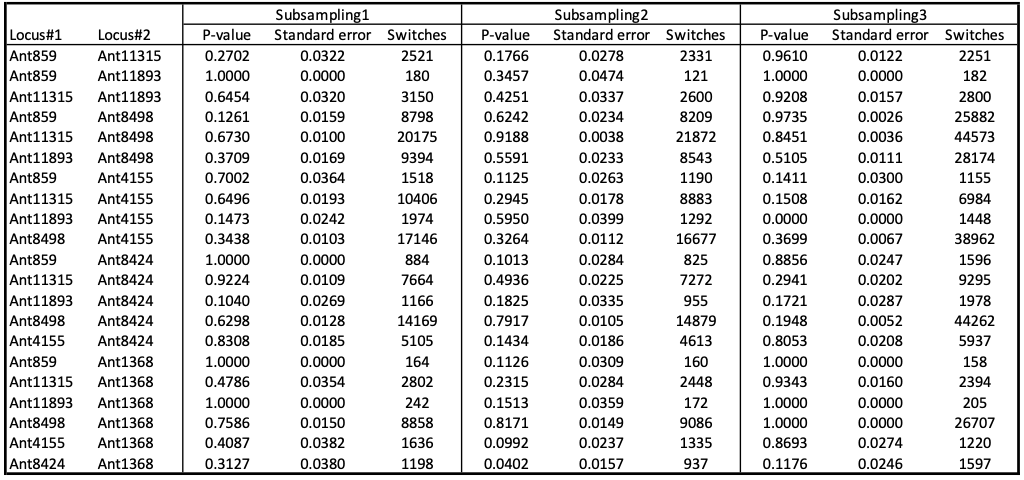


Table S3. Summary of data for each locus. *H_E(all)_* is expected heterozygosity when the complete dataset is used. *H_E(1)_*, *H_E(2)_*, and *H_E(3)_* are expected heterozygosities when calculated using subsampling 1, subsampling 2, and subsampling 3, respectively. *H_O_* is observed heterozygosity from the complete dataset.


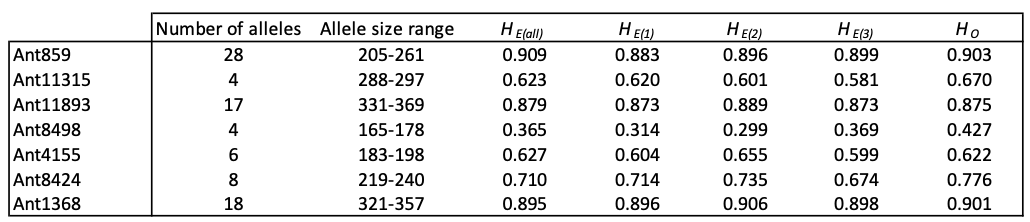


Table S4. P-values for comparisons of inter- and intra-year relatedness within samples for three subsampling schemes.


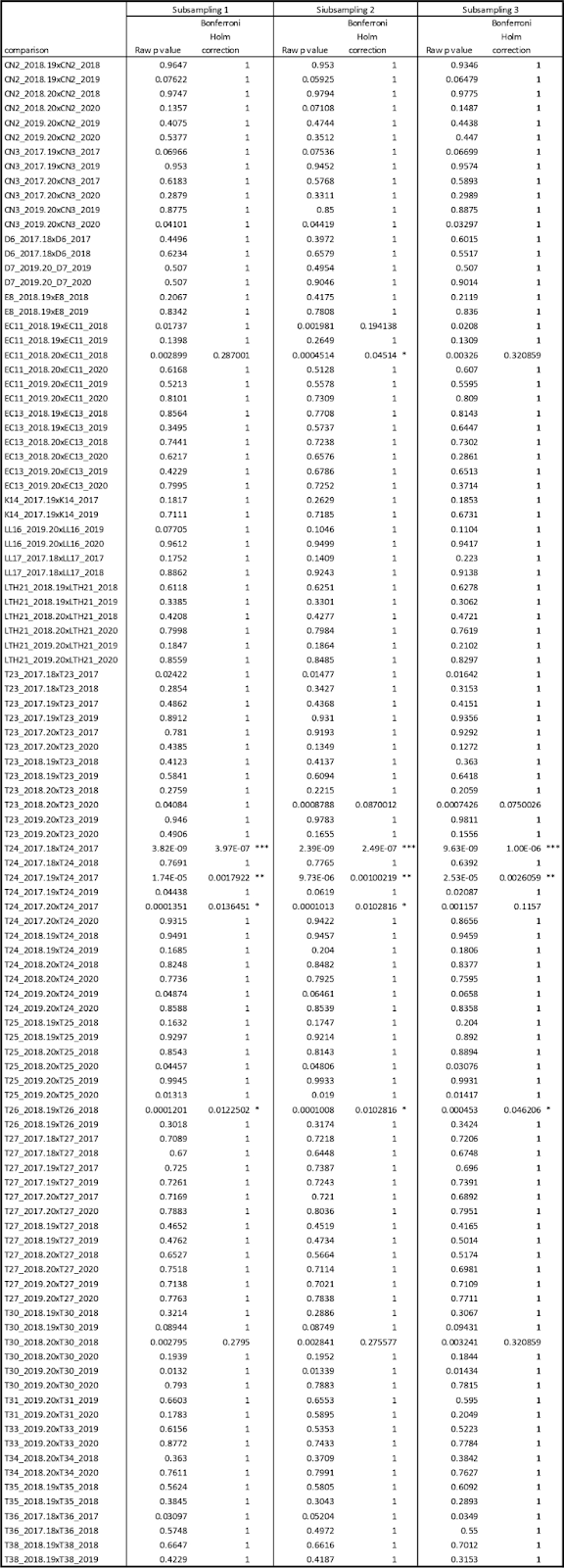


Table S5. List of samples in each relatedness category. Red text indicates samples that were split between categories in different subsampling schemes, listed here in the lower category. Numbers in parentheses are the lowest mean relatedness values from the three subsampling schemes.


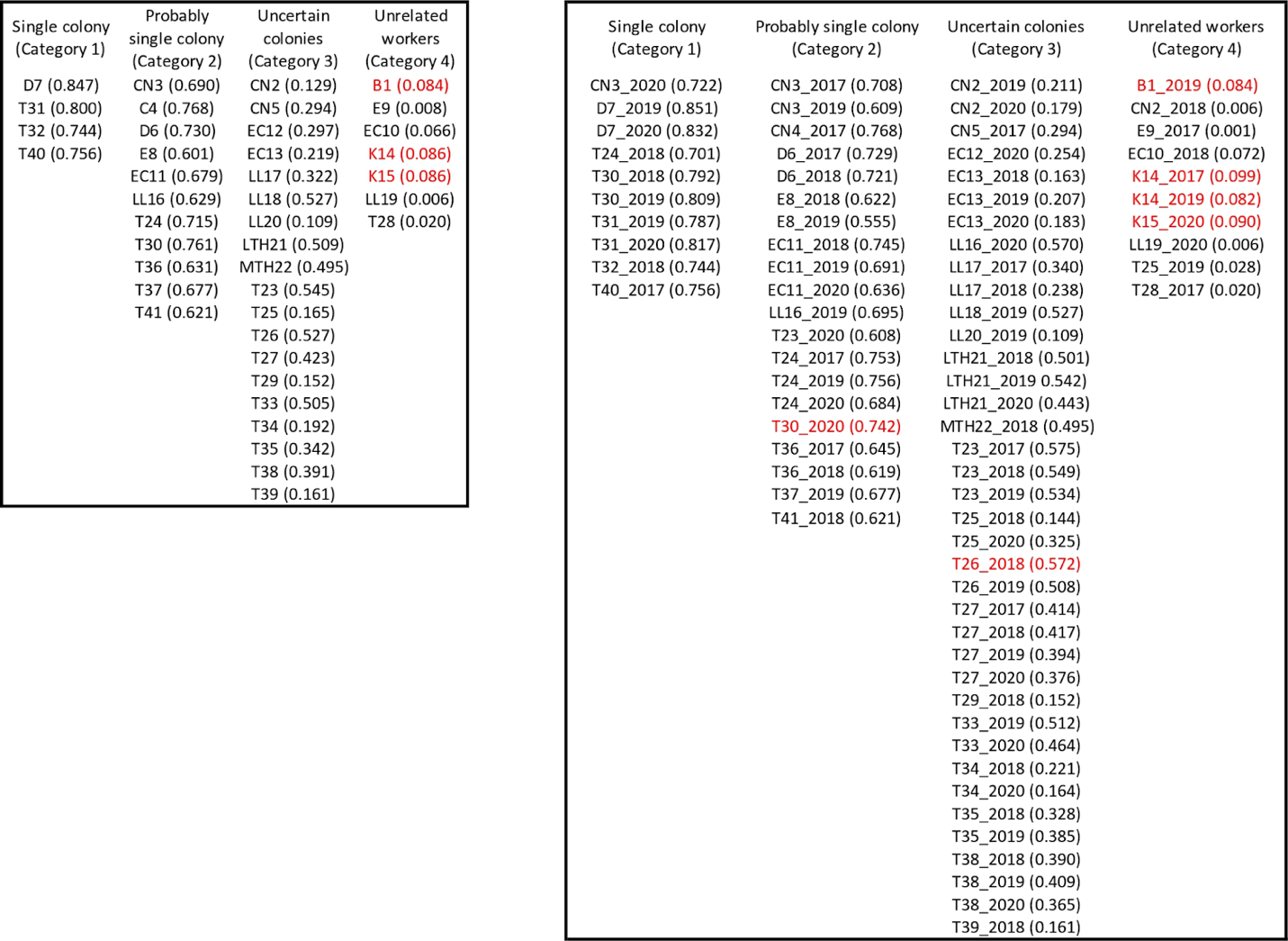


Table S6. Number of predicted queens and mates assigned to each sample. The column labeled Queen lists each predicted queen numbered 1 to n.The largest number in this column for each sample is the number of predicted queens for that sample. Number of offspring and number of mates refer to the number of offspring and mates, respectively, assigned to each queen.


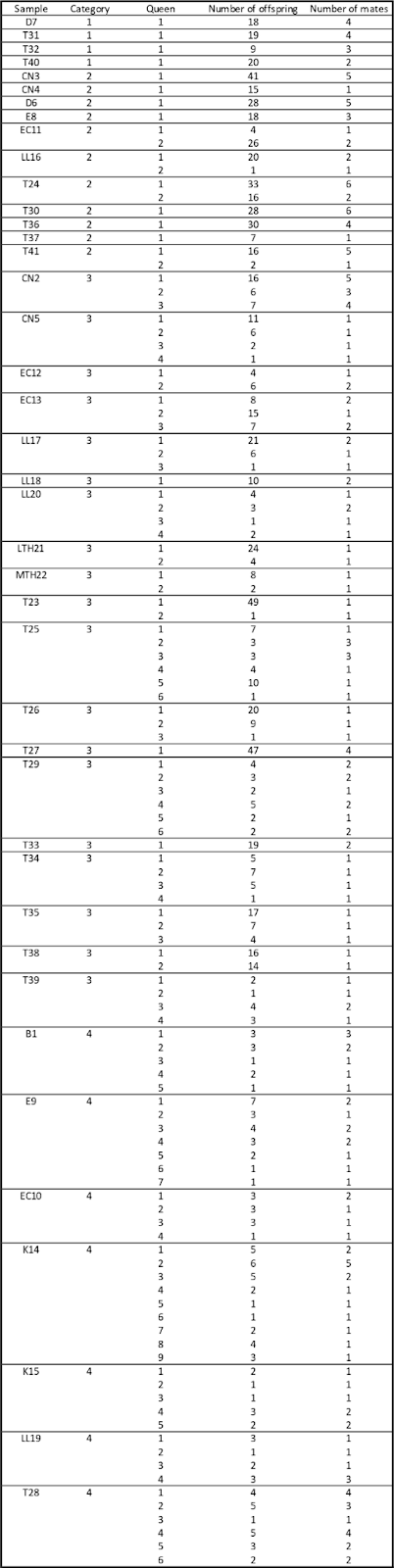


Table S7. Distances between nest sites of *C. goniodontus* in meters calculated from GPS coordinates. Labels are color coded according to relatedness category. Blue = single colonies (category 1), green = probably single colonies (category 2), yellow = uncertain colonies (category 3), red = unrelated groups of workers (category 4).


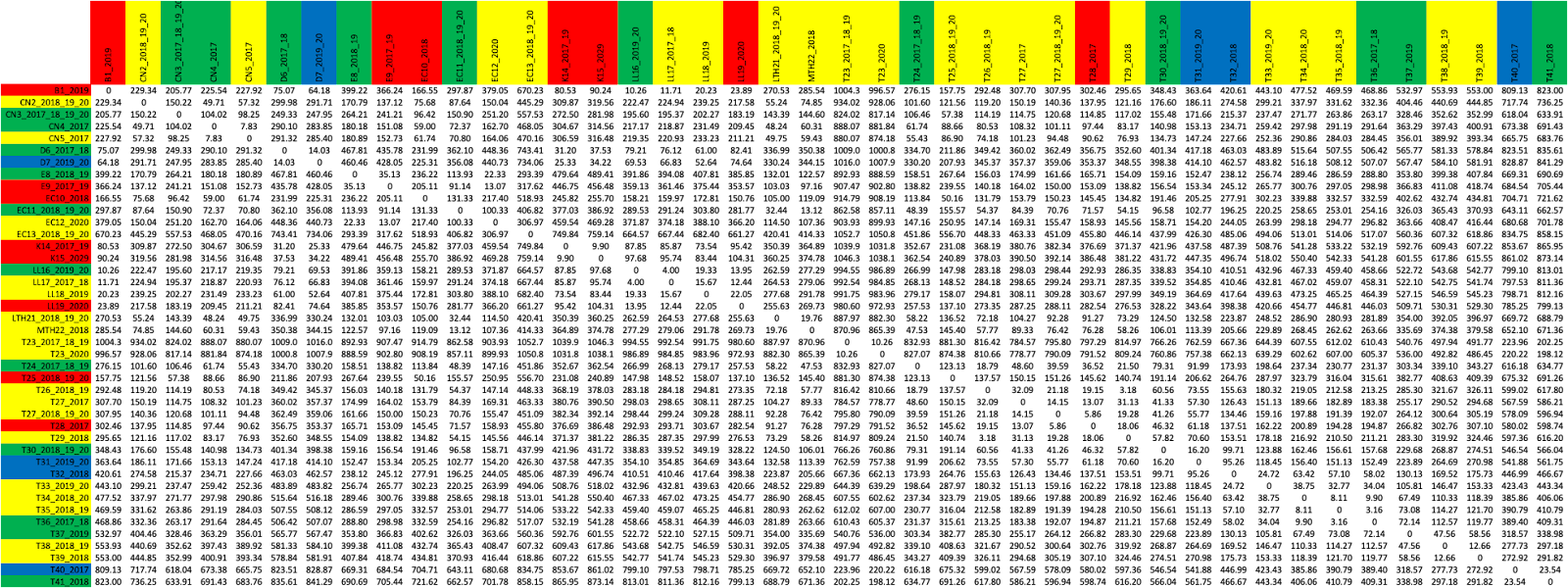


**Supplementary references**

[Raymond M, Rousset F (1995) GENEPOP (version 1.2): population genetics software for exact tests and ecumenicism. J Hered 86(3):248–249. https://doi.org/10.1093/oxfordjournals.jhered.a111573](https://www.zotero.org/google-docs/?0wuASC)

[Rousset F (2008) genepop’007: a complete re‐implementation of the genepop software for Windows and Linux. Mol Ecol Resour 8(1):103–106. https://doi.org/10.1111/j.1471-8286.2007.01931.x](https://www.zotero.org/google-docs/?0wuASC)

Weir BC, Cockerham CC (1984) Estimating F-statistics for the analysis of population structure. Evolution 38(6):1358–1370. https://doi.org/10.2307/2408641
